# Clinical Outcomes of ICSI Using Advanced Quantitative Phase Microscopy

**DOI:** 10.3390/bioengineering13020156

**Published:** 2026-01-28

**Authors:** Roni Rahav-Koren, Luba Nemerovsky, Yehudith Ghetler, Einat Haikin-Herzberger, Liat Salzer-Sheelo, Netanella Miller, Amir Wiser, Mattan Levi

**Affiliations:** 1 In Vitro Fertilization Unit, Department of Obstetrics and Gynecology, Meir Medical Center, Kfar Saba 4428163, Israelmattanlevi@gmail.com (M.L.); 2 Gray Faculty of Medical & Health Sciences, Tel Aviv University, Tel Aviv 6997801, Israel

**Keywords:** quantitative phase microscopy (QPM), sperm selection, intracytoplasmic sperm injection

## Abstract

This study evaluated the clinical outcomes associated with quantitative phase microscopy (QPM), an advanced 3D morphological sperm analysis technique using a virtual, stained model developed in our facility that indicates whether the inspected sperm should be selected for intracytoplasmic sperm injection (ICSI). We prospectively compared the clinical outcomes of three groups of cycles: (1) one with the QPM assessment, (2) one with conventional ICSI, without using QPM in the same patient, and (3) one with conventional ICSI cycles of an historical cohort of patients who did not undergo QPM. The outcomes of cycles using QPM vs. intracytoplasmic morphologically selected sperm injection (IMSI) cycles were also compared. A total of 1243 cycles were included. Pregnancy and delivery rates were higher among the QPM group compared to the standard ICSI group (56.2% vs. 18.1%, *p* = 0.04 and 50% vs. 9%, respectively, *p* = 0.02). Pregnancy and delivery rates were also higher among the QPM group compared to the IMSI group (56.2% vs. 16.6%, *p* = 0.01 and 50% vs. 16.6%, respectively, *p* = 0.03). No pregnancy complications were recorded in the QPM group. We conclude that QPM is a safe, non-invasive sperm selection technique for ICSI, with the potential to improve clinical pregnancy and delivery rates for couples with male infertility.

## 1. Introduction

Male infertility is a significant contributor to infertility worldwide. It is the sole cause in approximately 30% of cases [1,2] and is combined with other causes of infertility in up to 50% of cases [3]. Over the past few decades, studies have demonstrated a significant decline in sperm counts, globally [2,4,5,6,7]. Since intracytoplasmic sperm injection (ICSI) was introduced in 1992, it has become the most frequently used assisted reproductive technology (ART) worldwide, enabling biological parenthood for men with severe forms of male factor infertility [8,9]. Despite its advantages, and the use of definitive criteria for sperm selection [10,11], traditional manual sperm selection during ICSI is limited by the subjective morphological and motility assessment of the embryologist, and live birth rates remain low [12,13]

Sperm morphology has been widely investigated for its critical role in ART treatments [14,15,16]. Multiple studies have demonstrated a strong correlation between abnormal sperm morphology and impaired DNA integrity [14,17,18]; yet, sperm selection for injection based on DNA integrity is still challenging [19]. Thus, to improve clinical outcomes, novel interventions for sperm selection in ICSI have been proposed. Among these are intracytoplasmic morphologically selected sperm injection (IMSI). This sperm selection technique is based on the minor morphological criteria of six subcellular organelles using ultra-high magnification (6000×) microscopy. Although studies have demonstrated higher clinical pregnancy rates using IMSI [20,21], a Cochrane analysis could not support or refute its clinical use [12]. Other interventions described include hyaluronic acid sperm binding, magnetic-activated cell sorting, the zeta potential method and microfluids [22,23,24]. However, no method has been shown to clearly improve clinical pregnancy and live birth rates.

With the ongoing goal to improve the clinical outcomes of patients with male infertility based on ICSI, a new non-invasive, non-toxic technique for live and motile sperm selection, quantitative phase microscopy (QPM), has been described [25,26]. QPM uses an advanced optical imaging system to generate a 3D morphological sperm analysis by measuring the intracellular refractive index of each sperm cell selected for injection by the embryologist. The refractive index, which represents the cell’s dry mass, is then converted into a virtual stained model with criteria that indicate whether the sperm should be selected for injection [27,28]. The virtual stained model was found to be compliant with the 2021 WHO guideline for morphological assessment [11], and when compared to a chemical stain method, the repeatability of sperm measurements using QPM was significantly higher than that of the reference method [26]. In a preliminary study, Michailov et al. compared the clinical outcomes of 34 couples who used QPM to those of a control group of 42 couples who did not use QPM. They found significantly higher pregnancy rates (65% vs. 34%) among the QPM group [25]. To date, there are no other studies examining the clinical outcomes of QPM technology in sperm selection for ICSI. Furthermore, there are no data regarding live birth rates (LBRs) or comparing QPM to other sperm selection techniques.

This study aimed to achieve the following: 1. to compare the clinical outcomes of ICSI cycles with and without using QPM, and 2. to compare the clinical outcomes of ICSI cycles using QPM with IMSI cycles.

## 2. Materials and Methods

### 2.1. Study Design

This prospective cohort study included patients with male infertility who were recruited from the IVF clinic at Meir Medical Center from 15 January 2024 to 26 February 2025 to undergo morphological sperm analysis using QPM (which complies with the 2021 WHO morphological parameters [11]), following sperm collection and preparation as well as before ICSI. The inclusion criteria were patients intended for ICSI who provided signed informed consent, female patients, patients aged > 18 and ≤40, patients who had ≥4 fresh oocytes for injection, and those who had fresh ejaculated sperm with a concentration of ≥1 million/mL and ≥5% motility. The exclusion criteria were cycles for preimplantation genetic testing, frozen spermatozoa, surgically extracted spermatozoa, immotile sperm, severe oligozoospermia (≤1 million/mL following preparation), and frozen oocytes.

The clinical and laboratory data were compared between 3 groups of ICSI cycles: 1. the QPM study group, including ICSI cycles with QPM morphological sperm assessment and selection; 2. a self-control, conventional ICSI group, consisting of a previous or subsequent ICSI cycle of the same patients in the study group, without QPM; and 3. an historical cohort from a different patient population of conventional ICSI cycles who did not undergo QPM. To compare the 2 morphological assessment techniques of sperm selection for ICSI, the clinical outcomes of QPM were further compared to those of ICSI cycles using IMSI. All clinical and laboratory data were collected from electronic medical records from embryoscope (Vitrolife, Denmark) and LabProtector (LabProtector, Israel) data.

### 2.2. QPM Workflow and Sperm Selection Method

**Figure 1:**
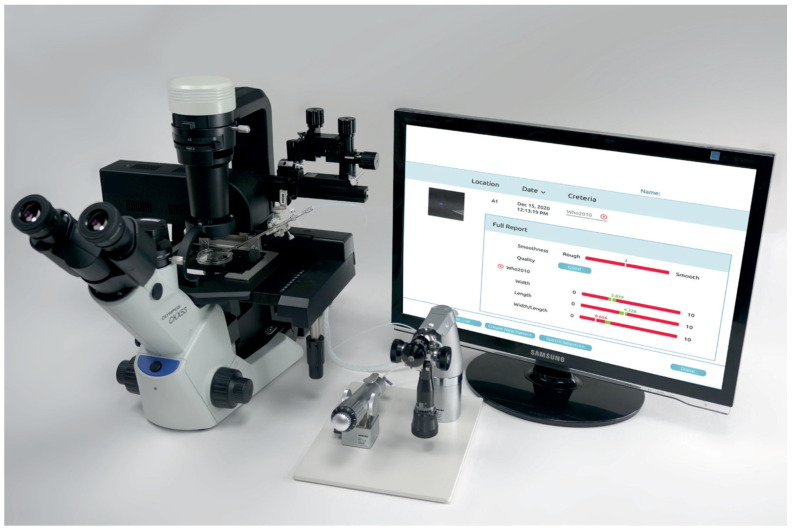
QPM system and a virtual stained model.

QPM uses an advanced optical imaging system (see the Supplementary Materials) to measure the intracellular refractive index of each sperm cell selected by the embryologist for injection. The refractive index is then converted into a virtual stained model, which is compliant with the 2021 WHO guidelines for morphological assessment (Figure 1). The virtual stained model obtained indicates whether the inspected sperm should be selected for injection. An example of a single sperm cell assessment using QPM is demonstrated in a video recording (Supplementary Material).

### 2.3. IVF Treatment Protocol

Study patients were treated with a flexible GnRH antagonist protocol, starting on day 2 or 3 of the menstrual cycle. Patients received daily injections of recombinant FSH (rFSH; Gonal F Merck Serono SA, Aubunne, Switzerland) and/or recombinant FSH and LH (Pergoveris, Merck Serono SA) and/or hMG (Menopur, Ferring SA, Sainet-Prex, Switzerland). When the dominant follicle was >13 mm in diameter, patients were treated with daily injections of GnRH antagonist (Ganirelix MSD or Cetrorelix, Merck Serono SA). When at least 3 follicles reached ≥17 mm in diameter, patients were treated with either 250 mcg hCG (Ovitrelle, Merck Serono SA) or 0.2 mg GnRHa to trigger ovulation. Ovum pick-up (OPU) was performed 36 h after ovulation triggering.

### 2.4. Frozen Embryo Transfer Protocols

Patients with normal ovulatory cycles were treated with a spontaneous frozen embryo transfer protocol and a single dose of 250 mcg hCG.

Patients with ovulatory dysfunction were treated with either an artificial endometrial preparation protocol or an aromatase inhibitor protocol.

### 2.5. Luteal Support

Patients undergoing fresh embryo transfer were treated with vaginal micronized progesterone (Endometrin 100 mg, TID or Crinone gel 8% BID) from day 1 following OPU.

Patients treated with GnRHa for ovulation triggering who underwent fresh embryo transfer were treated with repeated alternate-day doses of 0.1 mg GnRHa from day 3 following OPU, co-administered with vaginal micronized progesterone.

### 2.6. Sperm Preparation and Selection

Fresh semen was collected by masturbation after 2 to 5 days of sexual abstinence. Following liquefaction, the semen samples were then prepared for ICSI using gradient-density centrifugation with an isolate medium in two layers. After centrifugation at 500 *g* for 20 min, the pellet was aspirated, washed with multipurpose handling medium and centrifuged again. The final sperm suspension was then examined by the embryologist for sperm selection based on motility and morphology. In the QPM study group, each sperm cell selected by the embryologist was further analyzed by QPM, to determine which sperm cell to inject.

### 2.7. IMSI Procedure

IMSI was performed for patients with male factor or unexplained infertility and previous repeated IVF/ICSI failures. In our unit, the morphological assessment technique and sperm preparation is paid for by the patients. For shared decision making regarding whether to perform an IMSI cycle, patients were well informed about the technique, about the process of motile sperm organellar morphological examination for sperm selection for injection (IMSI), and about the inconclusive fertility outcomes reported in the literature to date. Informed consent was not required. Sperm preparation and selection for IMSI was performed at an external laboratory specializing in motile sperm organellar morphological examination.

### 2.8. Study Outcomes

The primary study outcomes were clinical pregnancy rate (CPR) and LBR among the QPM group, compared to historical control and self-control standard ICSI groups. The secondary outcomes were the CPR and LBR between the two morphologically based sperm selection techniques in the QPM and the IMSI groups.

### 2.9. Statistical Analysis

Since data regarding QPM are scarce, the sample size was calculated based on a previous preliminary study that included 34 cycles in the study group. For QPM vs. conventional ICSI comparison, 22 cycles per group were required, and for QPM vs. IMSI comparison, 25 cycles per group were required to provide a power of 80% at a 2-sided alpha level of 5%.

Data were analyzed using SPSS, version 24.0 for windows (IBM Corp., Armonk, NY, USA). For nominal parameters, data are presented as percentages and numbers, and for continuous variables, as the mean and standard deviation. *p*-values were considered significant at *p* < 0.05 and were calculated using χ^2^ or *t* tests. Live birth rates were compared using an exact McNemar test to account for within-patient pairing.

## 3. Results

Among 22 patients who provided signed informed consent, 18 met the inclusion criteria.

### 3.1. QPM Cycles vs. Self-Control and Historical Cohort Groups

The study population included 1224 ICSI cycles. In total, 18 cycles of 18 patients were recruited for the QPM group; 14 cycles of conventional ICSI without QPM assessment from 14 patients in the QPM group comprised the self-control group and 1192 conventional ICSI cycles constituted the historical control group (Table 1). The mean interval between ICSI cycles with the QPM assessment and conventional ICSI (the self-control group) was 247.5 ± 109.8 days. Women in the self-control group were older than in the QPM group (33.8 ± 1.2 vs. 34.1 ± 1.2 years, *p* = 0.04). The mean ages of the women in the QPM and the historical cohort groups were similar. The men in the QPM group demonstrated a statistical trend towards older age compared to the historical cohort group (36.9 ± 1.5 vs. 33.9 ± 0.2 years, *p* = 0.07). The mean age of the men in the QPM and self-control groups was similar.

Stimulation parameters including induction days, total gonadotropin dose, number of follicles ≥13 mm and progesterone on triggering day were similar between the groups. All patients in the historical control group were treated with an antagonist IVF protocol vs. 17 of 18 (92.8%) patients in the QPM group (*p* < 0.001). The mean number of cumulus oocyte complexes retrieved was higher in the QPM than in the self-control group (9 ± 5.3 vs. 7.9 ± 5.4, *p* = 0.02) and similar to that of the historical group (9 ± 5.3 vs. 10 ± 6.4, *p* = 0.52). The mean maturation rates (80.6% in the QPM, 78.4% in the self-control and 75.1% in the historical cohort groups) and semen parameters including volume concentration (31.8 ± 24 in the QPM, 41.8 ± 40 in the self-control and 23 ± 31.1 × 10^6^/mL in the historical cohort groups) and motility were similar between the groups (Table 1).

Fertility outcomes are depicted in Table 2. Mean fertilization rates were similar between the groups (66.4 ± 16.6, 64.4 ± 25.1 and 65.9 ± 23.9 in the QPM, self-control and historical cohort groups, respectively). The groups were similar in their KID-3 and KID-5 scores (Table 2) and in the rate of usable embryos per fertilization (61.7%, 58% and 50.9% in the QPM, self-control and historical cohort groups, respectively). The pregnancy rate was higher in the QPM group vs. the self-control group (56.2% vs. 18.1%, *p* = 0.04) and similar between the QPM group and the historical cohort group (56.2% vs. 44.3%, *p* = 0.3). The delivery rate was higher among the QPM group compared to the self-control group (50% vs. 9%, *p* = 0.02). Paired analysis was performed using an exact McNemar test among patients in the self-control and the QPM groups. Among 14 paired cycles, all discordant outcomes favored QPM: four patients achieved live birth using the QPM but not after conventional ICSI, whereas no cases of live birth were observed after conventional ICSI alone. This difference did not reach statistical significance (*p* = 0.12). Among the QPM deliveries, the mean delivery week was 38.8 ± 0.9 and the mean birthweight was 3183.4 ± 530.7 g. Except for one twin pregnancy with a term delivery, no pregnancy complications were recorded, including fetal anomalies, gestational diabetes, hypertensive disorders, intrauterine growth restriction, abnormal placentation and preterm birth. Of the eight deliveries, four were vaginal and four involved cesarean section. Among the nine pregnancies, one is ongoing.

### 3.2. QPM Cycles vs. IMSI Cycles

Eighteen ICSI cycles using the QPM device for sperm selection were compared to nineteen IMSI cycles. The mean female age was similar between groups (33.8 ± 1.2 vs. 32.4 ± 5.1, *p* = 0.6 in the QPM and IMSI groups, respectively) (Table 3). Men in the QPM group were older than those in the IMSI group (36.9 ± 1.5 vs. 33.8 ± 5.4, *p* = 0.02). Stimulation parameters including antagonist protocol, days of induction and follicles measuring ≥13 mm on the trigger day were similar between groups (Table 3). Also, the number of retrieved oocytes and the maturation rates were similar between the groups (9 ± 5.3 vs. 9.8 ± 4.6, *p* = 0.4 and 80.6% vs. 71.3%, *p* = 0.3 in the QPM vs. IMSI groups, respectively).

The fertility outcomes of the QPM and IMSI groups were compared (Table 4). The fertilization rates and number of usable embryos were similar between the groups (66.4% vs. 74.7%, *p* = 0.1 and 2.6 ± 2.4 vs. 2.3 ± 1.5, *p* = 0.5 for the QPM and IMSI groups, respectively). Pregnancy and delivery rates were higher among the QPM group (56.2% vs. 16.6%, *p* = 0.01 and 50% vs. 16.6%, *p* = 0.03).

## 4. Discussion

This study reports fertility outcomes after QPM, a non-invasive, novel technique for selecting live and motile sperm for injection, based on a 3D morphological sperm analysis. The outcomes of patients undergoing ICSI with QPM for sperm selection were compared to those from both conventional ICSI cycles within the same patient group and those from a historical group of conventional ICSI cycles in different patients who met the same inclusion criteria. The QPM cycles were then further compared to IMSI cycles.

Our findings demonstrate higher pregnancy and LBR among patients who had sperm selection for injection using QPM compared to the self-control, standard ICSI technique. These findings were further demonstrated in an exact McNemar test for LBR. All discordant paired outcomes favored QPM, with no instance in which live birth occurred following conventional ICSI but not QPM. While this difference did not reach statistical significance, likely due to the limited statistical power, the consistent directionality of the effect supports a potential clinical benefit of QPM for sperm selection. Furthermore, when compared to IMSI cycles, another technique for sperm selection for injection based on morphology, despite the older male age in the QPM group, higher pregnancy and LBR occurred. Except for one twin gestation with a term delivery, no pregnancy complications were recorded in the QPM group.

Methods for sperm selection for injection in cases of severe male factor are constantly being assessed, with the aim of improving the fertility outcomes achieved by conventional ICSI alone [19,22,29]. Although sperm selection during conventional ICSI is performed in accordance with strict guidelines [10,11], it depends on the subjective assessment of the embryologist. Difficulty in manually identifying high-quality sperm for ICSI has been described by Michailov et al. in their preliminary study on the QPM technique. A critical observation was that QPM classified only 40.9% of the embryologist-selected sperm as suitable for injection [25]. Our study results are similar to those of Michailov et al., who also demonstrated higher pregnancy rates among the QPM group. Currently, sperm selection techniques lack standardization. Approximately 20% of infertile males have an undetermined cause of infertility, and their treatment is limited due to lack of understanding of the frequency of general sperm defects [19]. Thus, certain techniques may be more successful than others according to the underlying etiology of male infertility, indicating a need for tailored treatment. This supports our findings that QPM was associated with higher pregnancy and delivery rates compared to conventional ICSI cycles among the same patients. Yet, compared to the historical cohort group of different patients meeting the same inclusion criteria, fertility outcomes were similar. Sperm DNA integrity has been consistently shown to be associated with fertility outcomes [30,31,32]. Nevertheless, techniques for sperm selection during preparation for injection based on DNA fragmentation are challenging. Sperm morphology has a crucial role and is strongly associated with sperm DNA integrity [14,17,18]. Thus, this study also aimed to compare two morphologically based techniques: QPM and IMSI. Higher pregnancy and delivery rates were found among the QPM group. IMSI has been widely investigated, with inconsistent results, and a recent Cochrane analysis did not support or refute its clinical use [12]. IMSI is also dependent on the subjective assessment of the embryologist, while QPM is a non-invasive technique that provides quantitative, objective evaluation of sperm cells, facilitating the embryologist’s decision of which sperm to inject.

The present study demonstrated that QPM for sperm selection is associated with higher pregnancy and delivery rates. In contrast to the study by Michailov et al. [25], it emphasized these results by including a self-control group and was the first to describe clinical information on LBR and pregnancy complications. Furthermore, this study compares two morphologically based sperm selection techniques.

Nevertheless, this study also had limitations. First was the small sample size of the QPM, the self-control and the IMSI groups relative to that of the historical cohort group. This imbalance between the QPM and historical cohort groups raises the potential for a type I error. Second was the combination of a prospective cohort with retrospective historical controls, which introduced potential selection and temporal biases. To minimize differences between the groups, all patients from the three study groups met the inclusion criteria, were ≤40 years, and had at least four fresh oocytes for injection. Furthermore, to minimize stimulation treatment effects, the majority of patients in the QPM and self-control groups, and all patients in the historical cohort, were treated with an antagonist IVF protocol. Also, the mean interval of approximately 8 months between the self-control and the QPM cycles could perhaps explain changes in patient characteristics, in terms of ovarian reserve, sperm quality, or laboratory protocols that could have influenced clinical outcomes. Nevertheless, despite the clinically insignificant differences in patients’ ages, the groups had similar characteristics, including male age, previous cycles, IVF protocol and stimulation and semen parameters.

The clinical relevance of QPM lies in its potential integration into routine ART laboratory practice without introducing additional invasive procedures, chemical exposure, or significant procedural burden. Unlike several sperm selection techniques that require prolonged handling times, invasive procedures or external laboratory processing, QPM is performed in real time on live, motile spermatozoa and builds directly upon the standard embryologist-driven ICSI practice.

Given the small number of QPM cycles, the generalizability of the present findings should be interpreted cautiously. However, several aspects of the study support their potential applicability to broader ART practice. First, all patients met commonly accepted inclusion criteria for ICSI, were treated using standard antagonist stimulation protocols, and underwent conventional semen preparation methods widely used in IVF laboratories. Second, QPM operates according to objective, quantitative morphological parameters that are compliant with WHO 2021 criteria, rather than center-specific or operator-dependent scoring systems. These characteristics suggest that, once validated in larger cohorts, QPM performance could be transferable to laboratories with embryologists with varying levels of experience.

Reproducibility is a critical consideration for the adoption of new laboratory technologies. In contrast to subjective morphology-based techniques such as IMSI, QPM provides standardized, quantitative measurements derived from optical phase data and automated algorithms. A previous study demonstrated the high repeatability of sperm measurements using QPM compared to conventional staining methods [26]. Together with its stain-free and non-destructive nature, this objective assessment may reduce inter-operator variability and enhance reproducibility both within and between IVF centers.

Nonetheless, the limited sample size of the QPM group underscores the need for larger, multicenter, randomized controlled trials to confirm clinical benefit and assess reproducibility across different laboratory environments. Also, tailoring procedures according to etiologies of undetermined male infertility is essential to standardizing the field of sperm selection techniques.

In conclusion, QPM is a safe, non-invasive technique with which to enhance sperm selection in ICSI, demonstrating potential for improving clinical pregnancy and delivery rates among patients with male infertility.

## Figures and Tables

**Table 1:** Patient and stimulation characteristics.

Variable	QPM	Paired Control (*p*-Value)	Historical Control (*p*-Value)
Cycles analyzed (*n*)	18	14	1192
Female age (years)	33.9 ± 1.2	34.2 ± 1.24 (0.04)	32.8 ± 0.2 (0.39)
Male age (years)	36.9 ± 1.5	35.1 ± 2.0 (0.1)	33.9 ± 0.2 (0.07)
Previous cycles	1.94 ± 0.93	1.25 ± 0.63 (0.64)	1.36 ± 0.05 (0.54)
Antagonist protocol, n (%)	18 (94.4)	13 (92.8) (0.85)	1192 (100) (<0.001)
Induction (days)	10.11 ± 0.36	11.14 ± 0.67 (0.1)	NA
Total dose (units)	3150.27 ± 291.1	3037.9 ± 464 (0.44)	NA
Progesterone on trigger day (ng/mL)	0.56 ± 0.1	0.63 ± 0.15 (0.46)	NA
Follicles ≥ 13 mm on trigger day	8.39 ± 4.9	7.23 ± 4.4(0.5)	8.79 ± 5 (0.73)
Cumulus oocyte complexes retrieved	9.06 ± 5.3	7.89 ± 5.4 (0.02)	10.02 ± 6.4 (0.52)
Mean maturation rate (%)	80.6 ± 19.07	78.49 ± 18.4 (0.7)	75.1 ± 22.1 (0.2)
Semen volume (mL)	3.27 ± 1.9	2.88 ± 1.9 (0.6)	3.15 ± 3.7 (0.81)
Sperm concentration (10^6^/mL)	31.81 ± 24	41.83 ± 40 (0.3)	23 ± 31.18 (0.2)
Mean sperm motility (%)	40.56 ± 21.25	47.21 ± 19.79 (0.3)	36.79 ± 21.7 (0.47)
Oocytes injected (n)	124	79	8973

Values are mean ± SD.

**Table 2:** Cycle outcomes.

Variable	QPM	Paired Control (*p*-Value)	Historical Control (*p*-Value)
Fertilization rate (%)	66.48 ± 16.6	64.43 ± 25.13 (0.78)	65.9 ± 23.94 (0.9)
KID-3 score	3.81 ± 0.21	4.18 ± 0.25 (0.27)	3.95 ± 0.13 (0.56)
KID-5 score	6.23 ± 0.32	5.09 ± 0.59 (0.12)	6.27 ± 0.22 (0.91)
Usable embryos per fertilization (%)	61.8 ± 36.8	58.03 ± 39.0 (0.7)	51.0 ± 29.30 (0.1)
Pregnancy rate per transfer, *n* (%)	9 (56.2)	2 (18.1) (0.04)	388 (44.3) (0.3)
LBR, *n* (%)	8 (50)	1 (9) (0.02)	NA

Values are mean ± SD, LBR live birth rate.

**Table 3:** QPM vs. IMSI cycles: patient and stimulation characteristics.

Variable	QPM	IMSI	*p*-Value
Cycles analyzed (*n*)	18	19	
Female age (years)	33.9 ± 1.2	32.4 ± 5.1	0.6
Male age (years)	36.9 ± 1.5	33.8 ± 5.5	0.02
Antagonist protocol, *n* (%)	17 (94.4)	19 (100)	0.2
Induction (days)	10.11 ± 0.36	10.26 ± 1.32	0.6
Progesterone on trigger day (ng/mL)	0.56 ± 0.1	0.83 ± 0.61	0.07
Follicles ≥ 13 mm on trigger day	8.39 ± 4.9	9.89 ± 4.68	0.3
Cumulus oocyte complexes retrieved	9.06 ± 5.3	9.89 ± 4.68	0.6
Mean maturation rate (%)	80.6 ± 19.07	71.3 ± 23	0.3
Oocytes injected (*n*)	124	118	

Values are mean ± SD.

**Table 4:** Fertility outcomes of QPM vs. IMSI cycles.

Variable	QPM	IMSI	*p*-Value
Fertilization rate (%)	66.4 ± 16.9	74.7 ± 15.3	0.1
Usable embryos	2.66 ± 2.4	2.36 ± 1.57	0.5
Pregnancy rate per transfer, n (%)	9 (56.2)	3 (16.6)	0.01
LBR, *n* (%)	8 (50)	3 (16.6)	0.03

Values are the mean ± SD. LBR: live birth rate.

## Data Availability

The original contributions presented in this study are included in the article/Supplementary Material. Further inquiries can be directed to the corresponding author.

## Supplementary Materials

The following supporting information can be downloaded at https://www.mdpi.com/article/10.3390/bioengineering13020156/s1: Video S1: A single sperm assessment using QPM video.

## Abbreviations

The following abbreviations are used in this manuscript:

CPR	Clinical pregnancy rate
ICSI	Intracytoplasmic sperm injection
IMSI	Intracytoplasmic morphologically selected sperm injection
LBR	Life birth rate
OPU	Ovum pick-up
QPM	Quantitative phase microscopy
